# The Accumulative Effect of Concentric-Biased and Eccentric-Biased Exercise on Cardiorespiratory and Metabolic Responses to Subsequent Low-Intensity Exercise: A Preliminary Study

**DOI:** 10.1515/hukin-2015-0115

**Published:** 2015-12-30

**Authors:** James Peter Gavin, Stephen Myers, Mark Elisabeth Theodorus Willems

**Affiliations:** 1 Department of Sport and Exercise Sciences, University of Chichester, United Kingdom; 2 Department of Sport and Physical Activity, Bournemouth University, United Kingdom

**Keywords:** muscle damage, eccentric exercise, exercise metabolism, low-intensity exercise, substrate oxidation

## Abstract

The study investigated the accumulative effect of concentric-biased and eccentric-biased exercise on cardiorespiratory, metabolic and neuromuscular responses to low-intensity exercise performed hours later. Fourteen young men cycled at low-intensity (~60 rpm at 50% maximal oxygen uptake) for 10 min before, and 12 h after: concentric-biased, single-leg cycling exercise (CON) (performed ~19:30 h) and eccentric-biased, double-leg knee extension exercise (ECC) (~06:30 h the following morning). Respiratory measures were sampled breath-by-breath, with oxidation values derived from stoichiometry equations. Knee extensor neuromuscular function was assessed before and after CON and ECC. Cardiorespiratory responses during low-intensity cycling were unchanged by accumulative CON and ECC. The RER was lower during low-intensity exercise 12 h after CON and ECC (0.88 ± 0.08), when compared to baseline (0.92 ± 0.09; p = 0.02). Fat oxidation increased from baseline (0.24 ± 0.2 g·min^−1^) to 12 h after CON and ECC (0.39 ± 0.2 g·min^−1^; p = 0.01). Carbohydrate oxidation decreased from baseline (1.59 ± 0.4 g·min^−1^) to 12 h after CON and ECC (1.36 ± 0.4 g·min^−1^; p = 0.03). These were accompanied by knee extensor force loss (right leg: −11.6%, p < 0.001; left leg: −10.6%, p = 0.02) and muscle soreness (right leg: 2.5 ± 0.9, p < 0.0001; left leg: 2.3 ± 1.2, p < 0.01). Subsequent concentric-biased and eccentric-biased exercise led to increased fat oxidation and decreased carbohydrate oxidation, without impairing cardiorespiration, during low-intensity cycling. An accumulation of fatiguing and damaging exercise increases fat utilisation during low intensity exercise performed as little as 12 h later.

## Introduction

Unaccustomed, high-intensity resistance exercise is associated with symptoms of exercise-induced muscle damage, particularly when involving eccentric contractions. The high eccentric forces produced during resistance exercise often result in acute force loss, muscle soreness and heightened effort perception during habitual activities in the subsequent hours and days. These persisting symptoms can impair endurance performance for lower- (Asp et al., 1998; Sargeant and Dolan, 1987) and upper-body exercise (Doncaster and Twist, 2012). Understanding the potential relationships between the physiological mechanisms of muscle damage is important to devise prevention and recovery strategies. A common approach to alleviate muscle damage symptoms after heavy training bouts is to prescribe low-intensity activity (Ahmaidi et al., 1996; Suzuki et al., 2004), aimed to improve muscle microcirculation.

Eccentric contractions may alter cardiorespiratory function during exercise-induced muscle damage, yet greater oxygen cost following duathlon (Calbet et al., 2001) and marathon events (Kyrolainen et al., 2000) suggests concentric-biased contractions also exert substantial ventilatory impairment. Elsewhere, bench-stepping exercise, involving both concentric and eccentric contractions, has resulted in elevated minute ventilation (V̇E), the respiratory exchange ratio (RER), and creatine kinase levels when cycling (15 min, at 80% maximal rate of oxygen uptake (V̇O2max)) 48 h later (Gleeson et al., 1995). However, oxygen uptake (V̇O2) was unaffected. Similar results have been observed at lower cycling workloads (60 and 80% peak V̇O2), with one-hundred countermovement jumps increasing perceptual and ventilatory responses, as well as impairing time-trial performance (Twist and Eston, 2009). The relationship between V̇E and perceived effort appears intensity-dependent, with the greater ventilatory responses at higher cycling workloads accompanied by higher perceived effort, 48 h after squatting exercise (Davies et al., 2009). Disrupted muscle microcirculation, occurring during exercise-induced muscle damage (Kano et al., 2005) elevates V̇E for subsequent exercise (Haouzi et al., 2004). A shortfall in oxygen delivery and utilisation may disturb muscle metabolism resulting in a shift to anaerobic metabolism, typified by increased lactate production (Braun and Dutto, 2003; Gleeson et al., 1995) and an emerging V̇O2 slow component at higher exercise intensities (Davies et al., 2008). It appears activity involving repeated, concentric and eccentric contractions may increase relative exercise-stress, and therefore influence exercise intensity and performance. This is important for recovery, when competing and/or training over repeated days, and may also present a novel strategy to manipulate substrate utilisation for subsequent exercise bouts.

Cardiorespiratory, metabolic and perceptual responses to exercise are intensity-dependent, as shown at moderate- (Molina and Denadai, 2011), heavy- (Schneider et al., 2007) and severe-intensities (Davies et al., 2009), as well as all-out effort (Twist and Eston, 2009). To-date, cardiorespiratory and metabolic responses for low-intensity exercise (at 50% V̇O2max), following exercise involving repeated, concentric and eccentric contractions have not been studied. Greater understanding of physiological responses during low-intensity exercise, with accompanying muscle fatigue and damage has relevance to i) athletes exercising for recovery, ii) those undertaking exercise rehabilitation and exercise-intolerant clinical groups, and iii) individuals aiming to manipulate substrate metabolism. For example, individuals using concurrent resistance exercise for training, and low-intensity aerobic exercise for recovery, would benefit from the knowledge of the acute effects of muscle fatigue and damage. Although 12 h after, is very acute for muscle damage, findings will provide insight into how early potential cardiovascular, respiratory and metabolic changes may emerge. In application, this is important to athletes performing morning training and low-intensity, evening training (such as swimmers, triathletes or track athletes), as well as overweight individuals targeting enhanced fat oxidation. Therefore, the study aim was to investigate the accumulative effect of concentric-biased and eccentric-biased exercise on cardiorespiratory and metabolic responses to low-intensity exercise in the hours after. The secondary aim was to examine the accompanying neuromuscular properties of the exercising muscle group, the knee extensors.

## Material and Methods

### Participants

Fourteen healthy men (mean ± SD; age, 22 ± 3 years, body height, 179 ± 6 cm, body mass, 76.4 ± 15.0 kg, body fat, 9.2 ± 2.5%) provided written informed consent to partake in the study. None had musculoskeletal and joint injury, all were recreationally active university students (exercise frequency, 2.9 ± 1.3 days per week) and not undertaking any form of lower-body resistance training during the testing period. Sample size estimation was based on data for MVC force loss (−19.8%, α level = 0.05, power = 0.80 (1 − β) (Faul et al., 2007). The research protocol was approved of by the University of Chichester Research Ethics Committee and conducted according to the Declaration of Helsinki.

### Procedures

Participants completed the following four experimental sessions: i) a familiarisation, ii) concentric-biased, single-leg cycling exercise (CON), iii) eccentric-biased, double-leg knee extension exercise (ECC), and iv) 12 h post low-intensity cycling. Single-leg cycling was performed to pre-fatigue the exercising leg, with concentric actions as part of a larger study (Gavin et al., 2015). Cardiorespiratory and metabolic responses were measured during 10 min of low-intensity, constant-load cycling prior to session two, and during session four. Responses were only measured 12 h post i) to examine a potential early-onset muscle damage, metabolic response and ii) to avoid influencing neuromuscular recovery as part of a related study (Gavin et al., 2015). Participants were instructed to refrain from heavy physical activity throughout the testing period, and to not to consume alcohol 24 h before each session. The familiarisation involved an incremental cycling trial to assess V̇O_2max_ and practice single-leg cycling. Session two occurred in the evening, at least 48 h later, and involved exhaustive, single-leg cycling in a 3 h fasted state, with the right leg (participant leg dominance: twelve (86%) right leg; two (14%) left leg). This was preceded by the two-leg, low-intensity cycling, a low carbohydrate evening meal (~3932 kJ: protein ~960 kJ, carbohydrate ~70 kJ, fat 2907 kJ) of three eggs and six bacon slices, and then a breakfast of two eggs the next day (Steensberg et al., 2002). Session three occurred early the following morning, and involved 100 single-leg, maximal eccentric knee extensions. Session four involved a 12 h retest of two-leg, low-intensity cycling exercise. Water was provided *ad libitum* during all sessions.

### Familiarisation

After collection of anthropometric data, subjects performed an incremental cycling test to volitional exhaustion on an electronically controlled ergometer (Excalibur Sport 925900, Lode, Groningen, the Netherlands). Participants cycled at a ~75 rpm cadence at 80 W for 60 s, with 30 W increments every 60 s, until a cadence of 50 rpm could not be maintained (Heil et al., 1995). The test measured the individual’s power at V̇O_2max_, which was used to determine workload for i) the low intensity cycling and, ii) the CON.

### Cardiorespiratory, metabolic and neuromuscular measures

Prior to CON and 12 h after ECC, two-leg low-intensity cycling was performed for 10 min at ~60 rpm on a friction-braked ergometer (Monark Ergomedic 824E, Varberg, Sweden) at 50% V̇O_2max_ (1.5 ± 0.2 kg; 116 ± 17 W). Participants were instructed to sustain a moderate-carbohydrate version of their habitual diet during the experimental period. Dietary information packs were provided to inform food preferences, and participants maintained food diaries which were monitored by the investigator on each visit. Respiratory responses were sampled throughout using a portable metabolic cart (Cosmed K4b^2^, Rome, Italy), with the heart rate (HR) measured continuously (Polar Electro Oy, Kempele, Finland). The metabolic cart was pre-calibrated to atmospheric air and gases of known concentration (O_2_ concentration, 15.12%; CO_2_ concentration, 5.03%). The turbine flow meter was calibrated with a 3 L calibration syringe (Model 5570, Hans Rudolph Inc., Kansas, USA) and with the investigator breathing at a regular, constant rhythm into a low-resistance face mask (Cosmed K4b^2^ User Manual). Breath-by-breath expired gas was collected during the low-intensity cycling, and averaged over 15 s periods for: tidal volume, V̇_E_, V̇O_2_, carbon dioxide output (V̇CO_2_), the RER and the HR. According to recommendations by Jeukendrup and Wallis (2005) for low-intensity exercise (40 to 50% V̇O_2max_), fat and carbohydrate oxidation values derived from respiratory measures (V̇O_2_ and V̇CO_2_) were modified using the following equations:

**Equation 1** Stoichiometry calculations proposed by Jeukendrup and Wallis (2005) for low-intensity exercise (40 to 50% V̇O_2max_).

$$\begin{array}{c} \text{Fat oxidation }(\text{g}\cdot {\text{min}}^{-1})\;1.695\times {\dot{\text{V}}\text{O}}_{2}-1.701\times {\dot{\text{V}}\text{CO}}_{2}-1.77\;n\\ \text{Carbohydrate oxidation }(\text{g}\cdot {\text{min}}^{-1})=4.344\times {\dot{\text{V}}\text{CO}}_{2}-3.061\times {\dot{\text{V}}\text{O}}_{2}-0.40\;n \end{array}$$

Where V̇O_2_ and V̇CO_2_ are presented in L·min^−1^ and oxidation rates in g·min^−1^; *n* = nitrogen contribution was assumed negligible. Oxidation estimations were based upon the assumption that total carbohydrate oxidation was derived from 50% plasma glucose and 50% muscle glycogen. Fat oxidation was derived from circulating long-chain fatty acids, low-density lipoprotein triglycerides and intramuscular triglycerides (Romijn et al., 1993). However, indirect calorimetry did not allow us to determine the proportional contribution of each (Jeukendrup and Wallis, 2005).

Knee extensor neuromuscular function was assessed before and after CON and ECC on a custom-built, strength-measurement chair. Seated and secured at the hips and chest, participants were positioned with hip and knee joints at 1.57 rad. The ankle was attached to a steel chain leading to the chair base and a calibrated s-beam load-cell. A DS7A electrical stimulator and a NeuroLog pulse generator (Digitimer Limited, Welwyn Garden City, UK) delivered electrical stimulation through two saline treated electrodes (9 × 18 cm) placed over the proximal and distal part of the thigh. A single, submaximal twitch was applied to the relaxed knee extensors. A warm up of three submaximal isometric knee extensions was followed by three MVC (3 to 5 s, with 2 min rest). Muscle soreness was assessed with the muscle-belly palpated under contraction, using a visual analogue scale (0, no pain; 10, extreme pain). Verbal encouragement and force-time feedback from the computer monitor were provided throughout maximal testing.

### Concentric-biased exercise

Between 19:00 and 20:00 h participants performed single-leg cycling exercise on a customised cycle ergometer (Monark Ergomedic 824E, Varberg, Sweden). The left crank was removed, and replaced with a platform supporting the left leg in a relaxed position. The right foot was secured into the pedal of the right crank. Maximal single-leg cycling workload was equated as 74% of maximal two-leg workload (at V̇O_2max_) from the familiarisation (Pernow and Saltin, 1971); all subsequent single-cycling workloads were derived from the single-leg maximal value and referred to hereafter. Single-leg cycling started at ~75 rpm with 20 min at 75% V̇O_2max_ (2.3 ± 0.3 kg; 178 ± 27 W), after which eight 90 s sprints were performed with a 1:1 work-to-rest ratio (workload decreased from 90 to 55% V̇O_2max_ at 5% decrements), before cycling at 85% V̇O_2max_ to volitional exhaustion (Pilegaard et al., 2002).

### Eccentric-biased exercise

The following morning (~06:30 h) participants performed maximal, eccentric knee extensions with the right leg, and then the left leg. Ten sets of ten eccentric contractions were performed (60 s rests between each set) using a pre-calibrated isokinetic dynamometer (Humac Norm, Cybex, NY, USA) (Byrne et al., 2001). Participants were seated and fastened in the strength testing chair with the hip joint at 1.57 rad. The lateral femoral epicondyle of the exercising leg was aligned to the gravity-corrected dynamometer and the ankle joint was secured to the lever arm. Participants were instructed to exert maximal force against the pre-programmed dynamometer lever arm, which moved at 1.57 rad·s^−1^ from full knee extension (0 rad) to full flexion (1.74 rad). Standardised verbal encouragement was provided throughout, with instantaneous visual feedback of torque-time traces from the dynamometer interface.

### Statistical analysis

Paired samples *t*-tests compared cardiorespiratory and metabolic responses for low-intensity cycling, and neuromuscular function, before and 12 h after CON and ECC (PASW Statistics 18.0, California, USA). Where data did not satisfy normal distribution (RER, fat oxidation and muscle soreness), Wilcoxon signed-rank tests were used. Cardiorespiratory and metabolic responses were compared for the final minute of low-intensity cycling, between responses before, and 12 h after CON and ECC. Cohen’s Effect sizes were calculated for meaningful differences (0.2 for small, 0.5 for moderate, and 0.8 for large). Data are presented as mean ± SD, with a significance level of *p* < 0.05 accepted.

## Results

### Cardiorespiratory measures

Cardiorespiration during low-intensity cycling was not affected by CON and ECC. Tidal volume was 1.58 ± 0.3 L at baseline and 1.63 ± 0.3 L 12 h after ECC (*p* = 0.6, *d* = 0.16); V̇_E_ was 35.2 ± 6.6 L·min^−1^ at baseline and 37.5 ± 5.7 L·min^−1^ at 12 h after ECC (*p* = 0.3, *d* = 0.36); V̇O_2_ was 1.46 ± 0.3 L·min^−1^ at baseline and 1.59 ± 0.2 L·min^−1^ 12 h after ECC (*p* = 0.1, *d* = 0.53); V̇CO_2_ was 1.36 ± 0.2 L·min^−1^ at baseline and 1.38 ± 0.2 L·min^−1^ 12 h after ECC (*p* = 0.7, *d* = 0.11); HR was 109 ± 16 b·min^−1^ at baseline and 111 ± 14 b·min^−1^ 12 h after ECC (*p* = 0.1, *d* = 0.18).

### Substrate oxidation

**Figure 1:**
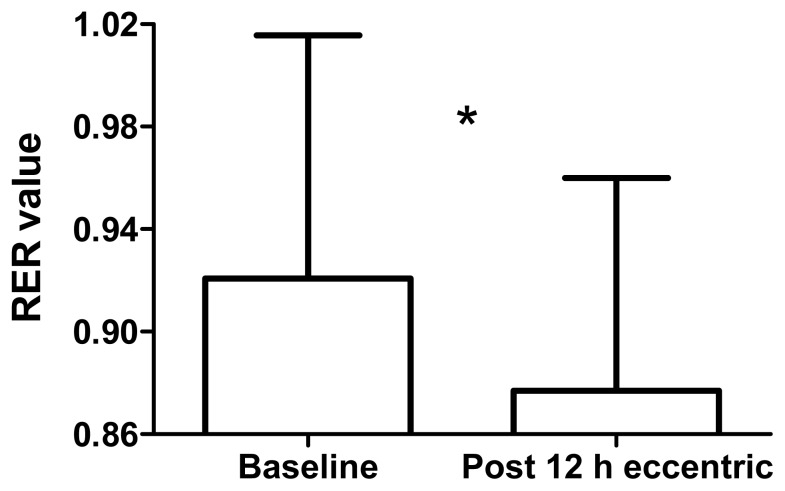
Respiratory exchange ratio (RER) values at baseline and 12 h after maximal, eccentric knee extensions with a prior concentric exercise. * Significant difference between time points, p < 0.05

**Figure 2:**
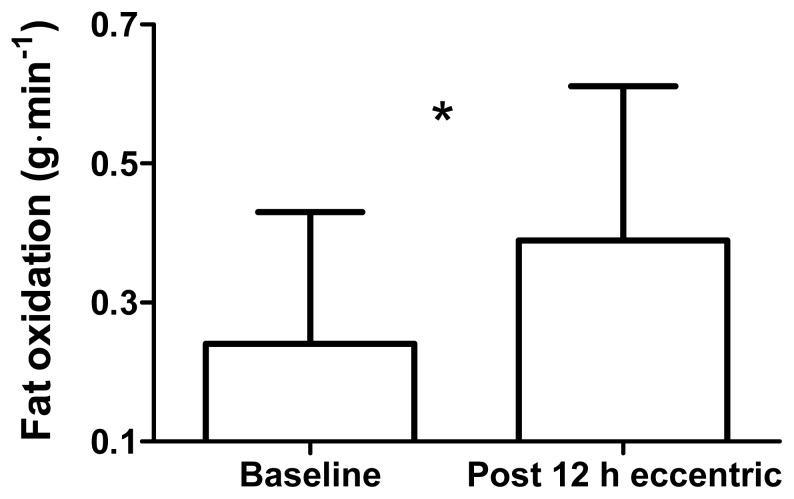
Fat oxidation values at baseline and 12 h after maximal, eccentric knee extensions with prior concentric exercise. Values are mean ± SD. * Significant difference between time points, p < 0.05

**Figure 3:**
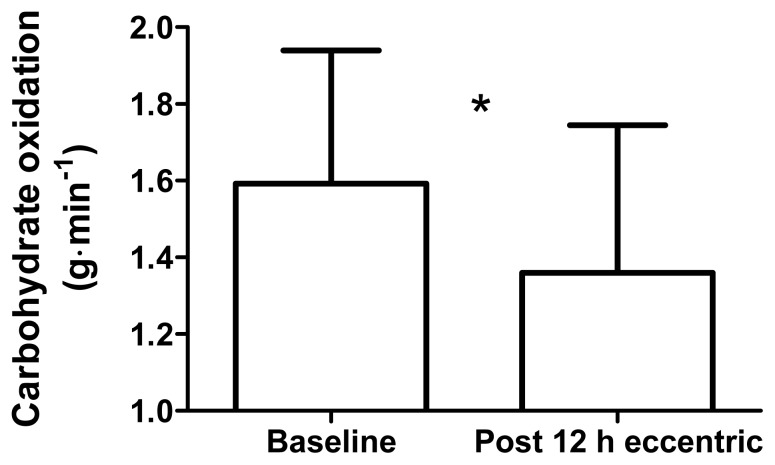
Carbohydrate oxidation at baseline and 12 h after maximal, eccentric knee extensions with prior concentric exercise. Values are mean ± SD. * Significant difference between time points, p < 0.05

During low-intensity cycling, the RER was lower 12 h after ECC (0.88 ± 0.08), than at baseline (0.92 ± 0.09; *p* = 0.02, *d* = 0.51; Figure 1). Fat oxidation was higher 12 h after ECC (0.39 ± 0.2 g·min^−1^), than at baseline (0.24 ± 0.2 g·min^−1^; *p* = 0.014, *d* = 0.72; Figure 2). Carbohydrate oxidation was lower 12 h after ECC (1.36 ± 0.4 g·min^−1^), than at baseline (1.59 ± 0.4 g·min^−1^; *p* = 0.03, *d* = 0.61; Figure 3).

### Neuromuscular measures

Twelve hours after ECC, knee extensor MVC decreased (right leg: *p* = 0.001, −11.6%, *d* = 0.93; left leg: *p* = 0.02, −10.6%, *d* = 0.72) and soreness increased (right leg: *p* = 0.0001, 2.5 ± 0.9, *d* = 1.56; left leg: *p* = 0.008, 2.3 ± 1.2, *d* = 0.99). The maximal rate of force decrease reduced after CON (right leg: only *p* = 0.05, *d* = 0.67), but single twitch force parameters were not significantly altered after CON and ECC (Table 1).

## Discussion

To characterise the relationship between cardiorespiratory and metabolic responses to exercise in a state of muscle damage, low-intensity cycling was performed before and after exhausting concentric, and then eccentric exercise. Our findings indicate that early mild-muscle damage of the knee extensors altered substrate metabolism, independent of cardiorespiratory measures, during low-intensity exercise. Peak damage occurs between 24 to 48 h, however, force loss results (−11.6% right leg; −10.6% left leg) indicate mild damage at 12 h according to Paulsen et al.’s (2012) criteria (<20% force loss). Despite unchanged cardiorespiratory function, a reduced RER was supported by increased fat oxidation and decreased carbohydrate oxidation. We hypothesised that knee extensor force loss 12 h after eccentric exercise would be associated with increased ventilation during low-intensity exercise. If muscle damage was manifest at 12 h, greater group III and IV afferent activation would increase ventilation. Force loss with muscle damage is associated with decreased cycling power output (Black and Dobson, 2012; Byrne et al., 2001) and treadmill running performance (Marcora and Bosio, 2007). Our eccentric exercise induced mild-muscle damage was evidenced by increased muscle soreness and force loss. Elsewhere, Davies et al. (2009) found similar force loss from squatting exercise after 30 min (−14%) and 48 h (−11%). We found the force loss during early muscle damage was not sufficient to alter effort perception for low-intensity cycling. Near peak muscle damage (~48 h), Twist and Eston (2009) observed increased effort perception and V̇E/V̇O2 (but unaltered V̇O2, HR and RER) during constant-load, submaximal cycling. At the same time-point, reduced time-trial performance was associated with greater effort perception.

Evidence supporting altered cardiorespiratory function when exercising in a muscle damaged state remains equivocal (Chen et al., 2007; Gleeson et al., 1995). We found no change in tidal volume, V̇E, V̇O2, V̇CO2 and HR during low intensity cycling 12 h after eccentric exercise, preceded by concentric exercise. This is not entirely surprising given previous mixed reports for greater exercise intensities, and with more severe eccentric exercise protocols. At higher cycling intensities, Davies et al. (2009) showed association between ventilation and effort perception for exercise above the gas exchange threshold. Participants underwent 6 min of moderate-, then severe-intensity cycling to volitional exhaustion, at baseline and 48 h preceding squatting exercise using 70% body weight. For moderate-intensity cycling, V̇E increased from 34.5 ± 5.0 to 36.3 ± 3.8 L·min^−1^ post 48 h, which was not dissimilar to our own observation from 35.2 ± 6.6 to 37.5 ± 5.7 L·min^−1^ 12 h later. These authors cited relation between respiratory responses and perceived exertion at severe-, but not moderate-intensities. This may suggest an intensity dependence on the manifestation of muscle damage symptoms when examining cardiorespiratory responses. Unaltered V̇O2 response in this study may be due to predominant recruitment of type I muscle fibres, as opposed to type II fibres.

Steady-state measures, such as V̇O2 and the RER, can indicate energy demands at a given work rate, but tell us little about oxygen delivery and utilisation. Participants cycled at an intensity of 50% V̇O2max, which was sufficiently low that inter-individual differences in V̇O2 and V̇CO2 were absent. Using the same isokinetic eccentric bout as our own, Molina and Denadai (2011) compared V̇O2 kinetics of untrained males during moderate-intensity cycling at either 50 or 100 rpm cadence. Cycling at 50 rpm, there was no change in phase II V̇O2 kinetics. Yet, at 100 rpm, a delay in V̇O2 became apparent at 24 h, suggesting exercise-induced impairment in oxygen delivery, in part, depends upon cadence. Awareness of the changes in cardiorespiratory and metabolic responses during the early-onset of muscle damage may impact the decisions of athletes, coaches and exercise scientists, when performing consecutive bouts of exercise. Such findings have importance for athletes including sailors, skiers and multi-event competitors, for whom it is common to perform repeat non-maximal/recovery exercise sessions over a single day.

The relative dietary contributions of carbohydrate and fat influence the RER at rest and during exercise. Interestingly, the RER decreased during low-intensity cycling in exercise-induced muscle damage at 12 h, indicating increased fat utilisation for the same given work rate as baseline. However, it is plausible that glycogen reduction from the concentric-biased exercise attenuated this decrease. Osborne and Schneider (2006) previously found glycogen depletion reduced the RER (depleted: 0.97; normal: 1.06) when performing 8 min of heavy-intensity, constant-load cycling. Lima-Silva et al. (2009) also found greater fat oxidation for cycling exercise with reduced glycogen, at workloads above the second lactate turnpoint. Such metabolic alterations during exercise warrant further research, particularly the approach of using prior, mixed concentric and eccentric exercise to promote fat metabolism. Yamanaka and associates (2012) used three separate, 120 s intense-cycling bouts (100 to 105% V̇O2peak), finding the RER decreased from bout one (1.05) to three (0.96). Therefore, our concentric-biased cycling exercise may have had a lasting effect on metabolic, but not cardiorespiratory responses. At baseline fat oxidation was 0.24 g·min^−1^, increasing to 0.39 g·min^−1^ 12 h into exercise-induced muscle damage. Whole-body, fat oxidation rates decline during high-intensity exercise (85% V̇O2max) as a result of muscle glycogen becoming the primary energy source (Romijn et al., 1993). In addition, as exercise-induced muscle damage disrupts glucose mobilisation in the young (Kirwan et al., 1992), our finding of greater fat (and reduced carbohydrate) contribution for low-intensity exercise during exercise-induced muscle damage, agrees with those at higher intensities. Tuominen et al. (1996) used the term ‘post marathon paradox’, to describe their observation of increased fat oxidation (by 55%) and decreased carbohydrate oxidation (by −43%) the morning following a marathon run. Similar to marathon running, which involves repeated, concentric and eccentric contractions of the knee extensors, the combination of our CON and ECC may partly explain the similar results.

Our experimental question was whether symptoms of exercise-induced muscle damage would become manifest for low-exercise intensity, in the following hours. This limited cardiorespiratory and metabolic measurement to an intensity below that of competitive and recreational activity, yet these findings have relevance to exercise during recovery. For example, bouts of fatiguing and damaging resistance exercise are often followed by low-intensity, aerobic exercise for recovery. The results also indicate that the accumulative effect of concentric and eccentric exercise elevates fat oxidation during acute, subsequent exercise. Optimising fat oxidation, this is relevant to i) encourage weight loss in the overweight/obese, and ii) act as a training stimulus for an athlete, that also promotes glycogen sparing. This study demonstrates that a protocol involving concentric-biased, single leg exercise, and then eccentric-biased, double leg exercise, did not alter cardiorespiratory responses during low-intensity cycling exercise, but did alter substrate oxidation. Respiratory measures remained unchanged following eccentric exercise, preceded by concentric-biased exercise; however, fat oxidation increased, while carbohydrate oxidation and the RER decreased. These findings suggest metabolic disruption may occur in exercise-induced muscle damage, without changing ventilation. Increased fat oxidation may be due to impaired insulin action with muscle damage; but this was not supported by cardiorespiratory responses. More likely was that the CON exercise reduced muscle glycogen levels prior to the eccentric exercise. These novel findings are particularly relevant to individuals commencing a new training regimen or period, as well as those combining endurance and resistance exercise in rehabilitation programmes. It appears undergoing a bout of muscle-damaging exercise with reduced muscle glycogen availability can raise fat utilisation, even for low exercise intensity, as short as 12 h later. In athletic cohorts this raised fat utilisation may be seen when incorporating plyometric and/or sprint sessions into habitual training (be it aerobic- or anaerobic-based). Whereas in recreational athletes, it may occur without a substantial eccentric component, but still be glycogen depleting (if long prolonged) leading to muscle damage. For example, when performing recreational endurance events (such as cycle sportives) or during adventure vacations (such as cross-country hiking or skiing).

The design may inform future research examining how combined, prior concentric and eccentric exercise can be used to optimise fat metabolism in sedentary and athletic populations.

As part of a larger study on the systemic neuromuscular effect of pre-fatiguing exercise on eccentric contractions, the within-subject design negated i) comparison with a control group (eccentric exercise only), and ii) low-intensity exercise immediately after eccentric exercise (potential to influence muscle damage). However, low-intensity cycling at baseline and 12 h responses demonstrate that our protocol altered metabolic responses. Our next study aims to introduce a control group to determine the impact of concentric-biased exercise on metabolic alterations during the time-course of exercise-induced muscle damage.

## Figures and Tables

**Table 1:** Knee extensor contractile parameters (right and left legs) during an evoked single twitch before, and after concentric-biased (CON) and eccentric-biased (ECC) exercise

Contractile parameter			Pre CON	Post CON	Pre ECC	Post ECC	Post 12 h
**Single twitch**	Contraction time (s)	Right leg	0.200 ± 0.043	0.184 ± 0.071	0.169 ± 0.035	0.163 ± 0.034	0.159 ± 0.034
		Left leg	0.204 ±0.054	0.158 ± 0.029	0.149 ± 0.047	0.167 ± 0.032	0.161 ± 0.037
	Half relaxation time (s)	Right leg	0.051 ± 0.008	0.044 ± 0.012	0.047 ± 0.01	0.034 ± 0.013	0.046 ± 0.014
		Left leg	0.050 ± 0.013	0.048 ± 0.016	0.054 ± 0.012	0.039 ± 0.012	0.047 ± 0.014
	Maximal rate of force development (N·s^−1^)	Right leg	841 ± 168	572 ± 173	784 ± 195	500 ± 158	711 ± 248
		Left leg	807 ± 210	721 ± 248	806 ± 254	625 ± 270	618 ± 182
	Maximal rate of force decrease (N·s^−1^)	Right leg	−557 ± 128	−419 ± 187	−560 ± 165	−393 ± 124	−558 ± 199
		Left leg	−522 ± 113	−565 ± 113 *	−546 ± 194	−451 ± 172	−450 ± 129

Data presented as mean ± SD.

* Significant difference between time points, p < 0.05.
